# Supplementary material for: Hybrid feature-selection and diversity-guided stacking framework for interpretable ensemble learning: Application to COVID-19 mortality prediction
Source: PLoS One. 2026 Apr 17;21(4):e0341198. doi: 10.1371/journal.pone.0341198 (PMC13089710; doi:10.1371/journal.pone.0341198)
Supplement: S1 File — This file contains supplementary tables, figures, and additional analyses including correlation matrices, model tuning parameters, performance summaries, feature importance results, interaction analyses, and SHAP outputs. (DOCX) [file pone.0341198.s001.docx]

**S1 Table. Correlation Matrix of Relationships Among Continuous and Binary Variables In The Dataset**

The correlation matrix provided presents the relationships between various continuous and binary

variables in our dataset as correlation coefficients.

- The correlation coefficient ranges from -1 to 1.
  - **1** indicates a perfect positive correlation, meaning as one variable increases, the other also increases.
  - **-1** indicates a perfect negative correlation, meaning as one variable increases, the other decreases.
  - **0** indicates no correlation, meaning the variables do not have a linear relationship.

Age Muscle.Pain Taste.Smell CKD O2sat.Ventilator NEUT

Age 1.00000000 -0.087297630 -0.052745099 0.085697833 -0.08510409 0.141066305

Muscle.Pain -0.08729763 1.000000000 0.053301588 -0.039536487 -0.01030588 -0.053708603

Taste.Smell -0.05274510 0.053301588 1.000000000 -0.016240351 -0.04076954 -0.022938683

CKD 0.08569783 -0.039536487 -0.016240351 1.000000000 0.05509670 0.034147421

O2sat.Ventilator -0.08510409 -0.010305880 -0.040769539 0.055096704 1.00000000 -0.117466780

NEUT 0.14106630 -0.053708603 -0.022938683 0.034147421 -0.11746678 1.000000000

NA. -0.01944164 0.003800895 0.008587861 -0.024871923 -0.03652063 -0.043917306

P 0.05179567 -0.081696603 -0.010086905 0.144909654 -0.09205876 0.036782446

FBS 0.03345589 0.087651114 0.032541462 0.019072638 -0.16613844 0.042879872

ESR 0.07712505 0.029402218 -0.030585684 0.036722767 -0.05506594 0.059668889

LACTATE 0.02557615 -0.020955318 0.003526150 0.014683144 -0.14797598 0.119036692

PROCALCITONIN -0.01508498 -0.044929287 0.077878183 0.038917993 -0.06142094 0.023934594

Ferritin 0.05917608 -0.019956622 -0.046571667 0.016323176 -0.06229512 0.114601665

TIBC -0.14410547 -0.032241224 -0.027006194 -0.008325373 0.04078179 -0.009086587

DDIMER 0.10318183 -0.112465018 0.024537041 0.012350827 -0.05542996 0.078612210

NA. P FBS ESR LACTATE PROCALCITONIN

Age -0.019441642 0.051795671 0.033455888 0.07712505 0.025576154 -0.01508498

Muscle.Pain 0.003800895 -0.081696603 0.087651114 0.02940222 -0.020955318 -0.04492929

Taste.Smell 0.008587861 -0.010086905 0.032541462 -0.03058568 0.003526150 0.07787818

CKD -0.024871923 0.144909654 0.019072638 0.03672277 0.014683144 0.03891799

O2sat.Ventilator -0.036520629 -0.092058763 -0.166138442 -0.05506594 -0.147975981 -0.06142094

NEUT -0.043917306 0.036782446 0.042879872 0.05966889 0.119036692 0.02393459

NA. 1.000000000 0.043894755 -0.047588478 -0.07989206 0.061960229 -0.06542993

P 0.043894755 1.000000000 -0.007200489 0.01308422 0.038986468 0.06260184

FBS -0.047588478 -0.007200489 1.000000000 0.09112815 0.221991623 0.06297464

ESR -0.079892056 0.013084219 0.091128149 1.00000000 -0.012787336 0.01876977

LACTATE 0.061960229 0.038986468 0.221991623 -0.01278734 1.000000000 0.19725957

PROCALCITONIN -0.065429934 0.062601840 0.062974638 0.01876977 0.197259570 1.00000000

Ferritin -0.061055930 -0.035792926 0.086378891 0.25754899 -0.015918349 0.02085211

TIBC 0.102302636 0.020261119 0.005719059 -0.35198131 0.006359734 0.01212575

DDIMER 0.012738894 0.097270382 -0.030800780 0.04277593 0.137653833 0.16263024

Ferritin TIBC DDIMER

Age 0.05917608 -0.144105466 0.10318183

Muscle.Pain -0.01995662 -0.032241224 -0.11246502

Taste.Smell -0.04657167 -0.027006194 0.02453704

CKD 0.01632318 -0.008325373 0.01235083

O2sat.Ventilator -0.06229512 0.040781792 -0.05542996

NEUT 0.11460167 -0.009086587 0.07861221

NA. -0.06105593 0.102302636 0.01273889

P -0.03579293 0.020261119 0.09727038

FBS 0.08637889 0.005719059 -0.03080078

ESR 0.25754899 -0.351981312 0.04277593

LACTATE -0.01591835 0.006359734 0.13765383

PROCALCITONIN 0.02085211 0.012125747 0.16263024

Ferritin 1.00000000 -0.335739662 0.12435005

TIBC -0.33573966 1.000000000 -0.11644540

DDIMER 0.12435005 -0.116445399 1.00000000

**S2 Table. P-Values Associated with The Correlation Matrix for Continuous and Binary Variables in The Dataset**

Age Muscle.Pain Taste.Smell CKD O2sat.Ventilator NEUT

Age NA 1.501125e-09 2.648160e-04 2.973310e-09 3.819912e-09 0.000000e+00

Muscle.Pain 1.501125e-09 NA 2.277830e-04 6.271513e-03 4.763368e-01 2.038367e-04

Taste.Smell 2.648160e-04 2.277830e-04 NA 2.617080e-01 4.824102e-03 1.128786e-01

CKD 2.973310e-09 6.271513e-03 2.617080e-01 NA 1.387693e-04 1.825299e-02

O2sat.Ventilator 3.819912e-09 4.763368e-01 4.824102e-03 1.387693e-04 NA 4.440892e-16

NEUT 0.000000e+00 2.038367e-04 1.128786e-01 1.825299e-02 4.440892e-16 NA

NA. 1.790643e-01 7.928113e-01 5.528633e-01 8.560709e-02 1.158301e-02 2.394453e-03

P 3.413086e-04 1.557576e-08 4.857570e-01 0.000000e+00 1.826512e-10 1.099962e-02

FBS 2.074375e-02 1.288597e-09 2.448877e-02 1.874587e-01 0.000000e+00 3.030996e-03

ESR 9.414461e-08 4.212538e-02 3.450519e-02 1.113025e-02 1.399701e-04 3.673738e-05

LACTATE 7.710620e-02 1.475398e-01 8.074823e-01 3.102330e-01 0.000000e+00 0.000000e+00

PROCALCITONIN 2.971768e-01 1.893761e-03 7.047397e-08 7.135641e-03 2.152016e-05 9.807938e-02

Ferritin 4.259203e-05 1.678209e-01 1.281540e-03 2.592827e-01 1.639283e-05 1.776357e-15

TIBC 0.000000e+00 2.583994e-02 6.195701e-02 5.650649e-01 4.811377e-03 5.300422e-01

DDIMER 8.713030e-13 6.217249e-15 8.990721e-02 3.933619e-01 1.263666e-04 5.300683e-08

NA. P FBS ESR LACTATE PROCALCITONIN

Age 1.790643e-01 3.413086e-04 2.074375e-02 9.414461e-08 7.710620e-02 2.971768e-01

Muscle.Pain 7.928113e-01 1.557576e-08 1.288597e-09 4.212538e-02 1.475398e-01 1.893761e-03

Taste.Smell 5.528633e-01 4.857570e-01 2.448877e-02 3.450519e-02 8.074823e-01 7.047397e-08

CKD 8.560709e-02 0.000000e+00 1.874587e-01 1.113025e-02 3.102330e-01 7.135641e-03

O2sat.Ventilator 1.158301e-02 1.826512e-10 0.000000e+00 1.399701e-04 0.000000e+00 2.152016e-05

NEUT 2.394453e-03 1.099962e-02 3.030996e-03 3.673738e-05 0.000000e+00 9.807938e-02

NA. NA 2.406877e-03 1.000217e-03 3.206319e-08 1.820178e-05 6.000102e-06

P 2.406877e-03 NA 6.187687e-01 3.658769e-01 7.034973e-03 1.488744e-05

FBS 1.000217e-03 6.187687e-01 NA 2.780576e-10 0.000000e+00 1.323484e-05

ESR 3.206319e-08 3.658769e-01 2.780576e-10 NA 3.768556e-01 1.945628e-01

LACTATE 1.820178e-05 7.034973e-03 0.000000e+00 3.768556e-01 NA 0.000000e+00

PROCALCITONIN 6.000102e-06 1.488744e-05 1.323484e-05 1.945628e-01 0.000000e+00 NA

Ferritin 2.408474e-05 1.335115e-02 2.226005e-09 0.000000e+00 2.712855e-01 1.495447e-01

TIBC 1.358469e-12 1.614270e-01 6.926814e-01 0.000000e+00 6.603038e-01 4.020417e-01

DDIMER 3.786660e-01 1.607470e-11 3.325486e-02 3.102618e-03 0.000000e+00 0.000000e+00

Ferritin TIBC DDIMER

Age 4.259203e-05 0.000000e+00 8.713030e-13

Muscle.Pain 1.678209e-01 2.583994e-02 6.217249e-15

Taste.Smell 1.281540e-03 6.195701e-02 8.990721e-02

CKD 2.592827e-01 5.650649e-01 3.933619e-01

O2sat.Ventilator 1.639283e-05 4.811377e-03 1.263666e-04

NEUT 1.776357e-15 5.300422e-01 5.300683e-08

NA. 2.408474e-05 1.358469e-12 3.786660e-01

P 1.335115e-02 1.614270e-01 1.607470e-11

FBS 2.226005e-09 6.926814e-01 3.325486e-02

ESR 0.000000e+00 0.000000e+00 3.102618e-03

LACTATE 2.712855e-01 6.603038e-01 0.000000e+00

PROCALCITONIN 1.495447e-01 4.020417e-01 0.000000e+00

Ferritin NA 0.000000e+00 0.000000e+00

TIBC 0.000000e+00 NA 6.661338e-16

DDIMER 0.000000e+00 6.661338e-16 NA

**S3 Table. Estimated Average Accuracy and Variance of the Sixteen Machine Learning Algorithms Across 100 Resamples**

Accuracy

Min. 1st Qu. Median Mean 3rd Qu. Max. NA's

GLM 0.6736527 0.7072437 0.7215569 0.7221487 0.7395210 0.7634731 0

LDA 0.6736527 0.7090513 0.7234695 0.7234333 0.7408526 0.7634731 0

lasso 0.6796407 0.7120431 0.7253731 0.7264838 0.7432836 0.7611940 0

ridge 0.6776119 0.7095808 0.7223881 0.7227180 0.7365269 0.7604790 0

elastic_net 0.6805970 0.7132183 0.7253731 0.7257972 0.7410448 0.7664671 0

KNN 0.8388060 0.8776119 0.8892216 0.8893286 0.9011976 0.9221557 0

NB 0.6846847 0.7223881 0.7313433 0.7351518 0.7514970 0.7791045 0

SVM 0.8383234 0.8655711 0.8776119 0.8764105 0.8865672 0.9129129 0

CART 0.5988024 0.6649254 0.6811393 0.6802070 0.6985075 0.7253731 0

NN 0.7155689 0.7373134 0.7492537 0.7529141 0.7707257 0.8083832 0

Kappa

Min. 1st Qu. Median Mean 3rd Qu. Max. NA's

GLM 0.3460392 0.4126772 0.4433229 0.4427093 0.4772888 0.5255852 0

LDA 0.3454748 0.4157950 0.4456997 0.4450576 0.4803869 0.5256194 0

lasso 0.3580384 0.4219649 0.4496134 0.4514308 0.4843940 0.5222476 0

ridge 0.3519845 0.4167147 0.4433026 0.4435285 0.4706052 0.5201322 0

elastic_net 0.3578582 0.4243359 0.4493970 0.4499947 0.4795152 0.5316747 0

KNN 0.6782742 0.7553677 0.7786958 0.7787599 0.8024164 0.8441717 0

NB 0.3647694 0.4411057 0.4594546 0.4668493 0.4997112 0.5554639 0

SVM 0.6774793 0.7310449 0.7552358 0.7528599 0.7733565 0.8255316 0

CART 0.2030911 0.3316516 0.3616270 0.3606800 0.3967955 0.4477692 0

NN 0.4295423 0.4742659 0.4968801 0.5054105 0.5402978 0.6162711 0

**Listing 1: Output of model resample statistics for base model types (GLM, LDA, lasso, ridge, elastic_net, KNN, NB, SVM, CART, NN)**

Accuracy

Min. 1st Qu. Median Mean 3rd Qu. Max. NA's

AdaBoost 0.8865672 0.9194030 0.9283582 0.9280748 0.9371727 0.9640719 0

C5.0 0.8862275 0.9124922 0.9223881 0.9217922 0.9341317 0.9610778 0

gbm 0.7904192 0.8353293 0.8477612 0.8487850 0.8649660 0.9044776 0

xgb 0.8802395 0.9044061 0.9134328 0.9135731 0.9222138 0.9461078 0

rf 0.8865672 0.9134328 0.9251497 0.9230751 0.9320404 0.9550898 0

treebag 0.8682635 0.8985075 0.9088212 0.9093248 0.9192220 0.9489489 0

Kappa

Min. 1st Qu. Median Mean 3rd Qu. Max. NA's

AdaBoost 0.7728003 0.8386636 0.8565899 0.8560320 0.8742444 0.9280198 0

C5.0 0.7727484 0.8249712 0.8448825 0.8435788 0.8682776 0.9221054 0

gbm 0.5806579 0.6707589 0.6954328 0.6974266 0.7294522 0.8088241 0

xgb 0.7608906 0.8087176 0.8269166 0.8272662 0.8445470 0.8922156 0

rf 0.7732455 0.8268996 0.8500090 0.8461348 0.8640969 0.9102762 0

treebag 0.7370926 0.7971144 0.8176621 0.8188025 0.8385908 0.8980496 0

**Listing 2: Output of model resample statistics for ensemble model types (AdaBoost, C5.0, gbm, xgb, rf, treebag)**

| 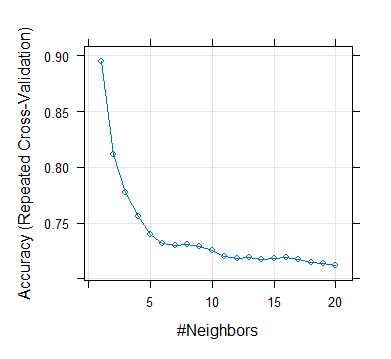  Knn | 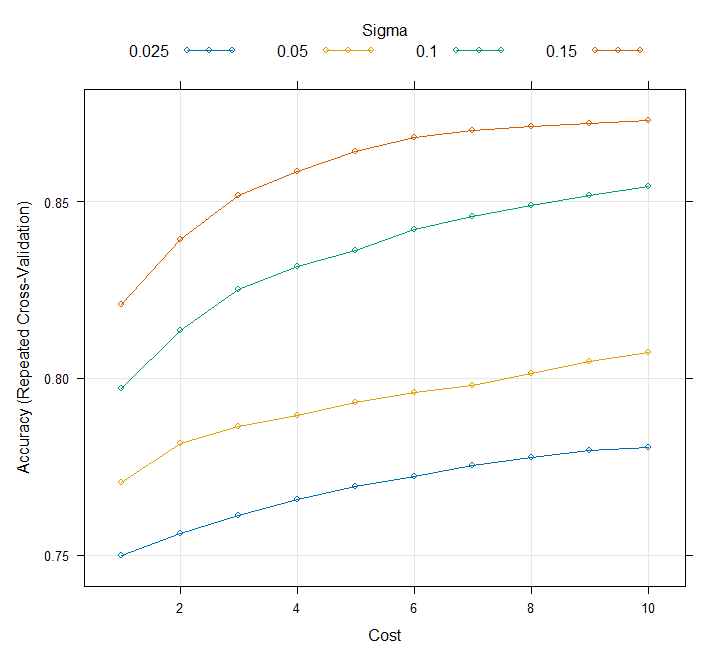  SVM |
| --- | --- |
| 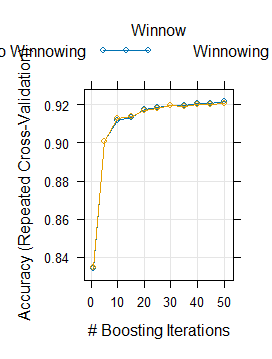  C5.0 | 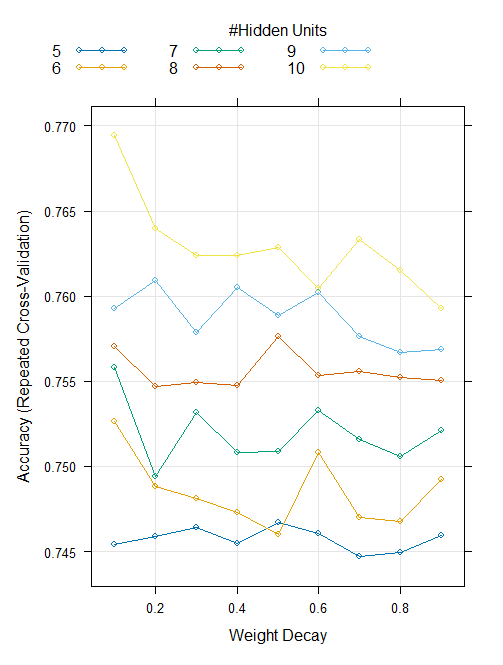  nnet |
| 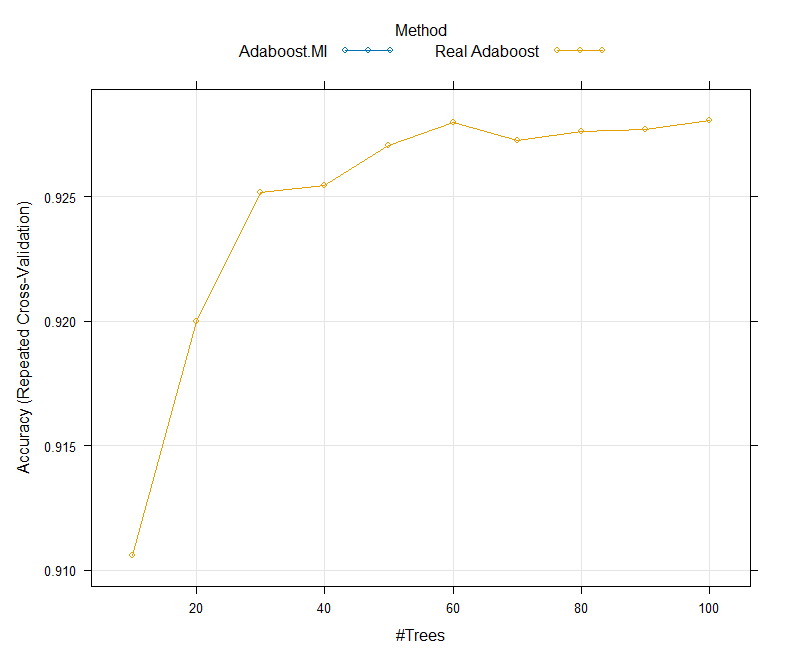  AdaBoost | 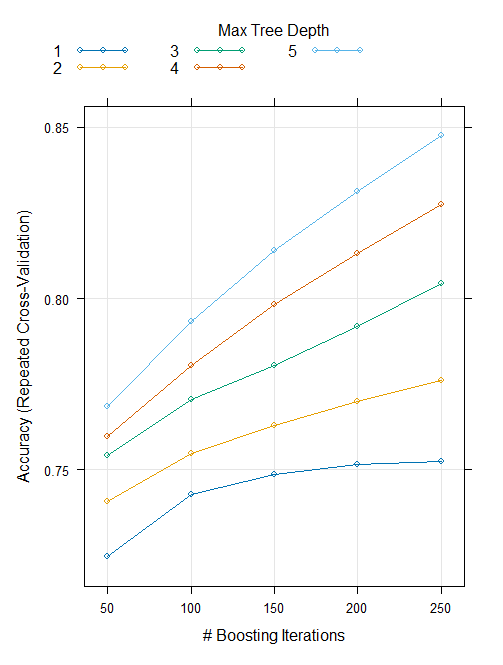  gbm |
| 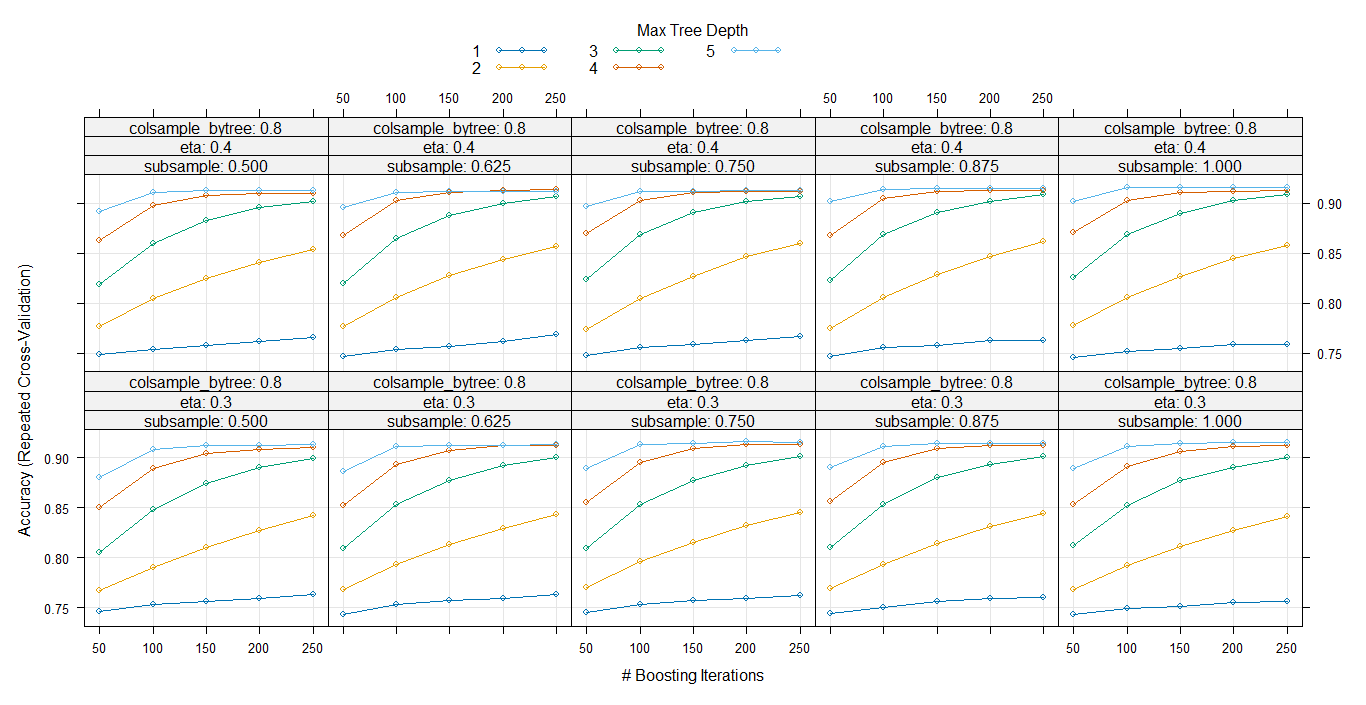  Extreme Gradient Boosting | |

**S1 Fig. Tuned Parameter Settings for the Selected Models**

**S4 Table. Pairwise Comparison Results Assessing Agreement and Disagreement Between Two Classifiers**

**- Disagreement measure**

predictions_glm predictions_lda predictions_glmnet predictions_ridge predictions_elastic_net predictions_nb

predictions_glm 0.00000000 0.01116539 0.032100488 0.03000698 0.034193999 0.1584089

predictions_lda 0.01116539 0.00000000 0.029309142 0.03140265 0.031402652 0.1612003

predictions_glmnet 0.03210049 0.02930914 0.000000000 0.05233775 0.003489184 0.1681786

predictions_ridge 0.03000698 0.03140265 0.052337753 0.00000000 0.053035590 0.1451500

predictions_elastic_net 0.03419400 0.03140265 0.003489184 0.05303559 0.000000000 0.1660851

predictions_nb 0.15840893 0.16120028 0.168178646 0.14515003 0.166085136 0.0000000

predictions_rpart 0.19818562 0.19120726 0.175854850 0.21423587 0.179344033 0.2686671

predictions_knn 0.28332170 0.28332170 0.281926029 0.27983252 0.284019539 0.2644801

predictions_svm 0.24633636 0.24354501 0.244940684 0.24145150 0.245638521 0.2330775

predictions_nnet 0.17096999 0.16817865 0.177948360 0.16887648 0.181437544 0.2121424

predictions.adaboost 0.40683880 0.40823447 0.406838800 0.40334962 0.408932310 0.3949756

predictions.C5.0 0.41172366 0.41451500 0.411723657 0.41381717 0.413817167 0.4166085

predictions.gbm 0.44801117 0.44940684 0.446615492 0.44033496 0.447313329 0.4459177

predictions.xgb 0.42986741 0.43126308 0.431263084 0.42219121 0.431960921 0.4110258

predictions.rf 0.40544313 0.40963015 0.405443126 0.40893231 0.407536636 0.4187020

predictions.treebag 0.43545010 0.43684578 0.435450105 0.42637823 0.436147941 0.4305652

predictions_rpart predictions_knn predictions_svm predictions_nnet predictions.adaboost predictions.C5.0 predictions.gbm

predictions_glm 0.1981856 0.2833217 0.2463364 0.1709700 0.4068388 0.41172366 0.4480112

predictions_lda 0.1912073 0.2833217 0.2435450 0.1681786 0.4082345 0.41451500 0.4494068

predictions_glmnet 0.1758548 0.2819260 0.2449407 0.1779484 0.4068388 0.41172366 0.4466155

predictions_ridge 0.2142359 0.2798325 0.2414515 0.1688765 0.4033496 0.41381717 0.4403350

predictions_elastic_net 0.1793440 0.2840195 0.2456385 0.1814375 0.4089323 0.41381717 0.4473133

predictions_nb 0.2686671 0.2644801 0.2330775 0.2121424 0.3949756 0.41660851 0.4459177

predictions_rpart 0.0000000 0.3321703 0.3119330 0.2393580 0.4180042 0.43126308 0.4535939

predictions_knn 0.3321703 0.0000000 0.1639916 0.2742498 0.4110258 0.41451500 0.4326588

predictions_svm 0.3119330 0.1639916 0.0000000 0.2316818 0.3977669 0.39706909 0.4403350

predictions_nnet 0.2393580 0.2742498 0.2316818 0.0000000 0.4075366 0.41102582 0.4515003

predictions.adaboost 0.4180042 0.4110258 0.3977669 0.4075366 0.0000000 0.14584787 0.3886950

predictions.C5.0 0.4312631 0.4145150 0.3970691 0.4110258 0.1458479 0.00000000 0.4047453

predictions.gbm 0.4535939 0.4326588 0.4403350 0.4515003 0.3886950 0.40474529 0.0000000

predictions.xgb 0.4410328 0.4173064 0.4152128 0.4235869 0.3845080 0.40614096 0.1200279

predictions.rf 0.4207955 0.4096301 0.4033496 0.4103280 0.1242149 0.05792045 0.3956734

predictions.treebag 0.4466155 0.4145150 0.4138172 0.4333566 0.3886950 0.39357990 0.1939986

predictions.xgb predictions.rf predictions.treebag

predictions_glm 0.4298674 0.40544313 0.4354501

predictions_lda 0.4312631 0.40963015 0.4368458

predictions_glmnet 0.4312631 0.40544313 0.4354501

predictions_ridge 0.4221912 0.40893231 0.4263782

predictions_elastic_net 0.4319609 0.40753664 0.4361479

predictions_nb 0.4110258 0.41870202 0.4305652

predictions_rpart 0.4410328 0.42079553 0.4466155

predictions_knn 0.4173064 0.40963015 0.4145150

predictions_svm 0.4152128 0.40334962 0.4138172

predictions_nnet 0.4235869 0.41032798 0.4333566

predictions.adaboost 0.3845080 0.12421493 0.3886950

predictions.C5.0 0.4061410 0.05792045 0.3935799

predictions.gbm 0.1200279 0.39567341 0.1939986

predictions.xgb 0.0000000 0.39986043 0.1912073

predictions.rf 0.3998604 0.00000000 0.3942777

predictions.treebag 0.1912073 0.39427774 0.0000000

**- Yule's or Q-statistic**

Yule's coefficient serves as a quantitative measure of agreement between two raters. When its value approaches zero, it indicates a significant degree of disagreement between the two evaluators. The Q-statistic operates within the range of the correlation coefficient, defined as -1 ≤ Q ≤ 1. Thus, Q values that are closer to 1 suggest that the two measures exhibit a high level of concordance, whereas values approaching -1 indicate that the predictions made by the two models are in substantial opposition to one another. When Q values are nearer to 0, it signifies a very weak association between the two raters.

predictions_glm predictions_lda predictions_glmnet predictions_ridge predictions_elastic_net predictions_nb

predictions_glm 0.000000000 0.999727411 0.997666246 0.99815525 0.99728921 0.926942351

predictions_lda 0.999727411 0.000000000 0.998120725 0.99802377 0.99776129 0.924150381

predictions_glmnet 0.997666246 0.998120725 0.000000000 0.99332201 0.99998287 0.912796573

predictions_ridge 0.998155246 0.998023774 0.993322012 0.00000000 0.99320192 0.938199408

predictions_elastic_net 0.997289212 0.997761292 0.999982868 0.99320192 0.00000000 0.916254697

predictions_nb 0.926942351 0.924150381 0.912796573 0.93819941 0.91625470 0.000000000

predictions_rpart 0.870089204 0.880902448 0.901890576 0.84214240 0.89733698 0.719923635

predictions_knn 0.677959356 0.679028995 0.677845414 0.68042397 0.67326813 0.702335066

predictions_svm 0.775742382 0.783268452 0.775240003 0.78028750 0.77499379 0.780214575

predictions_nnet 0.907899960 0.911601129 0.898007218 0.90941995 0.89346828 0.842070630

predictions.adaboost 0.137039315 0.132968916 0.122827251 0.12956129 0.11666941 0.077118644

predictions.C5.0 0.138126335 0.126693400 0.126046923 0.10427534 0.11968245 -0.001210476

predictions.gbm 0.009449544 0.004987495 0.006498195 0.02930591 0.00673884 -0.071388822

predictions.xgb 0.046575884 0.042348257 0.026278461 0.06368707 0.02739575 0.036840106

predictions.rf 0.162005630 0.143292252 0.149516711 0.11985338 0.14323596 -0.030820491

predictions.treebag 0.036602717 0.032293958 0.024879635 0.06197295 0.02566296 -0.044384357

predictions_rpart predictions_knn predictions_svm predictions_nnet predictions.adaboost predictions.C5.0 predictions.gbm

predictions_glm 0.870089204 0.677959356 0.77574238 0.90789996 0.13703932 0.138126335 0.009449544

predictions_lda 0.880902448 0.679028995 0.78326845 0.91160113 0.13296892 0.126693400 0.004987495

predictions_glmnet 0.901890576 0.677845414 0.77524000 0.89800722 0.12282725 0.126046923 0.006498195

predictions_ridge 0.842142401 0.680423965 0.78028750 0.90941995 0.12956129 0.104275336 0.029305913

predictions_elastic_net 0.897336984 0.673268127 0.77499379 0.89346828 0.11666941 0.119682454 0.006738840

predictions_nb 0.719923635 0.702335066 0.78021457 0.84207063 0.07711864 -0.001210476 -0.071388822

predictions_rpart 0.000000000 0.517800043 0.58693172 0.79322756 0.07916008 0.038474217 -0.015841008

predictions_knn 0.517800043 0.000000000 0.90721215 0.69736385 -0.01810865 0.012704174 0.001413721

predictions_svm 0.586931716 0.907212147 0.00000000 0.80322760 0.03213563 0.087112314 -0.061182640

predictions_nnet 0.793227562 0.697363845 0.80322760 0.00000000 0.10995760 0.122079226 -0.024413266

predictions.adaboost 0.079160077 -0.018108652 0.03213563 0.10995760 0.00000000 0.921983243 0.138049836

predictions.C5.0 0.038474217 0.012704174 0.08711231 0.12207923 0.92198324 0.000000000 0.088313184

predictions.gbm -0.015841008 0.001413721 -0.06118264 -0.02441327 0.13804984 0.088313184 0.000000000

predictions.xgb -0.009858713 0.002592505 -0.01207389 0.05939559 0.05048068 -0.022947057 0.959358289

predictions.rf 0.084154150 0.024699554 0.03388706 0.11562497 0.94538121 0.990395668 0.125083020

predictions.treebag -0.018550365 0.047072703 0.02764424 0.02844841 0.06553435 0.088780219 0.862889871

predictions.xgb predictions.rf predictions.treebag

predictions_glm 0.046575884 0.162005630 0.03660272

predictions_lda 0.042348257 0.143292252 0.03229396

predictions_glmnet 0.026278461 0.149516711 0.02487964

predictions_ridge 0.063687069 0.119853380 0.06197295

predictions_elastic_net 0.027395753 0.143235963 0.02566296

predictions_nb 0.036840106 -0.030820491 -0.04438436

predictions_rpart -0.009858713 0.084154150 -0.01855036

predictions_knn 0.002592505 0.024699554 0.04707270

predictions_svm -0.012073888 0.033887059 0.02764424

predictions_nnet 0.059395594 0.115624967 0.02844841

predictions.adaboost 0.050480679 0.945381211 0.06553435

predictions.C5.0 -0.022947057 0.990395668 0.08878022

predictions.gbm 0.959358289 0.125083020 0.86288987

predictions.xgb 0.000000000 -0.004961958 0.85736279

predictions.rf -0.004961958 0.000000000 0.06751431

predictions.treebag 0.857362789 0.067514310 0.00000000

**- Cohen's statistic**

Cohen's statistic is a metric that evaluates the probability of agreement between two classifiers attributable to chance or coincidence. Cohen's kappa can assume negative values, indicating varying degrees of agreement. A kappa value of 1 signifies complete agreement between the classifiers, while a value of 0 suggests that the observed agreement is solely due to chance. Conversely, a negative value indicates that the level of agreement is less than what would be anticipated by random chance.

| predictions_glm predictions_lda predictions_glmnet predictions_ridge predictions_elastic_net predictions_nb  predictions_glm 0.000000000 0.975792539 0.930107641 0.93447081 0.925644287 0.6459128636  predictions_lda 0.975792539 0.000000000 0.936241618 0.93148575 0.931773529 0.6400957648  predictions_glmnet 0.930107641 0.936241618 0.000000000 0.88528426 0.992386172 0.6223020835  predictions_ridge 0.934470808 0.931485745 0.885284257 0.00000000 0.883914924 0.6726693682  predictions_elastic_net 0.925644287 0.931773529 0.992386172 0.88391492 0.000000000 0.6276621760  predictions_nb 0.645912864 0.640095765 0.622302084 0.67266937 0.627662176 0.0000000000  predictions_rpart 0.570317565 0.585794460 0.617449707 0.53258053 0.610351041 0.4001596081  predictions_knn 0.366975092 0.367713960 0.367135267 0.36924286 0.363556604 0.3842484833  predictions_svm 0.445985537 0.452942753 0.446374068 0.45186589 0.445834331 0.4523387243  predictions_nnet 0.626954165 0.633377389 0.610313368 0.62899716 0.603214497 0.5221593826  predictions.adaboost 0.056846238 0.055110522 0.050819786 0.05366936 0.048216095 0.0313011325  predictions.C5.0 0.059571136 0.054535155 0.054210061 0.04463552 0.051428769 -0.0005005421  predictions.gbm 0.004223456 0.002229396 0.002896332 0.01306493 0.003006546 -0.0306092014  predictions.xgb 0.019877197 0.018066545 0.011170190 0.02718821 0.011652562 0.0153891201  predictions.rf 0.069350503 0.061122874 0.063833639 0.05085393 0.061086927 -0.0125491083  predictions.treebag 0.015911517 0.014035620 0.010781713 0.02695221 0.011129452 -0.0186081020  predictions_rpart predictions_knn predictions_svm predictions_nnet predictions.adaboost predictions.C5.0 predictions.gbm  predictions_glm 0.5703175646 0.3669750924 0.445985537 0.62695416 0.056846238 0.0595711359 0.0042234557  predictions_lda 0.5857944596 0.3677139597 0.452942753 0.63337739 0.055110522 0.0545351549 0.0022293965  predictions_glmnet 0.6174497071 0.3671352675 0.446374068 0.61031337 0.050819786 0.0542100612 0.0028963322  predictions_ridge 0.5325805283 0.3692428605 0.451865888 0.62899716 0.053669360 0.0446355193 0.0130649328  predictions_elastic_net 0.6103510407 0.3635566041 0.445834331 0.60321450 0.048216095 0.0514287692 0.0030065460  predictions_nb 0.4001596081 0.3842484833 0.452338724 0.52215938 0.031301132 -0.0005005421 -0.0306092014  predictions_rpart 0.0000000000 0.2586991251 0.299327824 0.47820931 0.032497782 0.0163345551 -0.0070666029  predictions_knn 0.2586991251 0.0000000000 0.614919297 0.38255445 -0.007217279 0.0052681399 0.0006119208  predictions_svm 0.2993278239 0.6149192972 0.000000000 0.47470326 0.012851625 0.0363739504 -0.0259999614  predictions_nnet 0.4782093133 0.3825544488 0.474703265 0.00000000 0.045379363 0.0524370050 -0.0108283845  predictions.adaboost 0.0324977823 -0.0072172785 0.012851625 0.04537936 0.000000000 0.6269526020 0.0569973713  predictions.C5.0 0.0163345551 0.0052681399 0.036373950 0.05243700 0.626952602 0.0000000000 0.0373126203  predictions.gbm -0.0070666029 0.0006119208 -0.025999961 -0.01082838 0.056997371 0.0373126203 0.0000000000  predictions.xgb -0.0041779024 0.0010771013 -0.004964505 0.02534811 0.019944120 -0.0091940059 0.7151903269  predictions.rf 0.0355316748 0.0101576483 0.013863258 0.04903120 0.679194975 0.8543936302 0.0526984031  predictions.treebag -0.0080147017 0.0200276768 0.011624134 0.01231974 0.026265354 0.0368659640 0.5450617016  predictions.xgb predictions.rf predictions.treebag  predictions_glm 0.019877197 0.069350503 0.015911517  predictions_lda 0.018066545 0.061122874 0.014035620  predictions_glmnet 0.011170190 0.063833639 0.010781713  predictions_ridge 0.027188214 0.050853933 0.026952207  predictions_elastic_net 0.011652562 0.061086927 0.011129452  predictions_nb 0.015389120 -0.012549108 -0.018608102  predictions_rpart -0.004177902 0.035531675 -0.008014702  predictions_knn 0.001077101 0.010157648 0.020027677  predictions_svm -0.004964505 0.013863258 0.011624134  predictions_nnet 0.025348114 0.049031204 0.012319741  predictions.adaboost 0.019944120 0.679194975 0.026265354  predictions.C5.0 -0.009194006 0.854393630 0.036865964  predictions.gbm 0.715190327 0.052698403 0.545061702  predictions.xgb 0.000000000 -0.001976842 0.533404476  predictions.rf -0.001976842 0.000000000 0.027650843  predictions.treebag 0.533404476 0.027650843 0.000000000 |
| --- |
|  |
| \|  \| \| --- \| \|  \| |

**- Double-fault measure**

The Double_Fault measure quantifies the disagreement between two sets of predictions. A Double_Fault value of 1 would mean that the two sets of predictions completely disagree with each other. This means that for every observation, if one prediction is correct, the other one is incorrect. Conversely, a Double_Fault value of 0 would indicate perfect agreement between the two sets of predictions, meaning that they are identical. When the Double_Fault value is above 0.8, it suggests that the two sets of predictions are significantly different and disagree on a large number of observations.

| predictions_glm predictions_lda predictions_glmnet predictions_ridge predictions_elastic_net predictions_nb  predictions_glm 0.0000000 0.8569435 0.8527565 0.8534543 0.8527565 0.8387997  predictions_lda 0.8569435 0.0000000 0.8527565 0.8527565 0.8527565 0.8381019  predictions_glmnet 0.8527565 0.8527565 0.0000000 0.8499651 0.8534543 0.8353105  predictions_ridge 0.8534543 0.8527565 0.8499651 0.0000000 0.8499651 0.8381019  predictions_elastic_net 0.8527565 0.8527565 0.8534543 0.8499651 0.0000000 0.8353105  predictions_nb 0.8387997 0.8381019 0.8353105 0.8381019 0.8353105 0.0000000  predictions_rpart 0.8374040 0.8374040 0.8353105 0.8339149 0.8353105 0.8248430  predictions_knn 0.8248430 0.8241452 0.8227495 0.8241452 0.8227495 0.8227495  predictions_svm 0.8262387 0.8255408 0.8234473 0.8262387 0.8234473 0.8262387  predictions_nnet 0.8394976 0.8381019 0.8360084 0.8387997 0.8360084 0.8318214  predictions.adaboost 0.8025122 0.8025122 0.8018144 0.8004187 0.8018144 0.8004187  predictions.C5.0 0.8087927 0.8080949 0.8066992 0.8066992 0.8066992 0.8046057  predictions.gbm 0.8164689 0.8157711 0.8143754 0.8150733 0.8143754 0.8094906  predictions.xgb 0.8108863 0.8101884 0.8080949 0.8094906 0.8080949 0.8073971  predictions.rf 0.8101884 0.8094906 0.8080949 0.8080949 0.8080949 0.8066992  predictions.treebag 0.8073971 0.8073971 0.8066992 0.8073971 0.8066992 0.8025122  predictions_rpart predictions_knn predictions_svm predictions_nnet predictions.adaboost predictions.C5.0 predictions.gbm  predictions_glm 0.8374040 0.8248430 0.8262387 0.8394976 0.8025122 0.8087927 0.8164689  predictions_lda 0.8374040 0.8241452 0.8255408 0.8381019 0.8025122 0.8080949 0.8157711  predictions_glmnet 0.8353105 0.8227495 0.8234473 0.8360084 0.8018144 0.8066992 0.8143754  predictions_ridge 0.8339149 0.8241452 0.8262387 0.8387997 0.8004187 0.8066992 0.8150733  predictions_elastic_net 0.8353105 0.8227495 0.8234473 0.8360084 0.8018144 0.8066992 0.8143754  predictions_nb 0.8248430 0.8227495 0.8262387 0.8318214 0.8004187 0.8046057 0.8094906  predictions_rpart 0.0000000 0.8192603 0.8164689 0.8304257 0.8039079 0.8094906 0.8143754  predictions_knn 0.8192603 0.0000000 0.8339149 0.8276343 0.7990230 0.8060014 0.8115841  predictions_svm 0.8164689 0.8339149 0.0000000 0.8290300 0.7990230 0.8060014 0.8080949  predictions_nnet 0.8304257 0.8276343 0.8290300 0.0000000 0.8018144 0.8080949 0.8108863  predictions.adaboost 0.8039079 0.7990230 0.7990230 0.8018144 0.0000000 0.8227495 0.8039079  predictions.C5.0 0.8094906 0.8060014 0.8060014 0.8080949 0.8227495 0.0000000 0.8046057  predictions.gbm 0.8143754 0.8115841 0.8080949 0.8108863 0.8039079 0.8046057 0.0000000  predictions.xgb 0.8108863 0.8066992 0.8046057 0.8087927 0.7997209 0.7997209 0.8450803  predictions.rf 0.8087927 0.8066992 0.8053036 0.8073971 0.8241452 0.8429867 0.8066992  predictions.treebag 0.8053036 0.8066992 0.8039079 0.8039079 0.7990230 0.8011165 0.8325192  predictions.xgb predictions.rf predictions.treebag  predictions_glm 0.8108863 0.8101884 0.8073971  predictions_lda 0.8101884 0.8094906 0.8073971  predictions_glmnet 0.8080949 0.8080949 0.8066992  predictions_ridge 0.8094906 0.8080949 0.8073971  predictions_elastic_net 0.8080949 0.8080949 0.8066992  predictions_nb 0.8073971 0.8066992 0.8025122  predictions_rpart 0.8108863 0.8087927 0.8053036  predictions_knn 0.8066992 0.8066992 0.8066992  predictions_svm 0.8046057 0.8053036 0.8039079  predictions_nnet 0.8087927 0.8073971 0.8039079  predictions.adaboost 0.7997209 0.8241452 0.7990230  predictions.C5.0 0.7997209 0.8429867 0.8011165  predictions.gbm 0.8450803 0.8066992 0.8325192  predictions.xgb 0.0000000 0.8025122 0.8269365  predictions.rf 0.8025122 0.0000000 0.8018144  predictions.treebag 0.8269365 0.8018144 0.0000000 |
| --- |
|  |
| \|  \| \| --- \| |

**S5 Table. Results of Feature Importance Analysis**

feature importance.05 importance importance.95 permutation.error

1 Age 1.8729017 1.8992806 1.930456 0.2367713

2 NEUT 1.3098321 1.3453237 1.347242 0.1677130

3 P 1.2954436 1.3165468 1.325659 0.1641256

4 Ferritin 1.2115108 1.2685851 1.276259 0.1581465

5 O2sat.Ventilator 1.2258993 1.2374101 1.246043 0.1542601

6 LACTATE 1.1405276 1.1870504 1.217746 0.1479821

7 PROCALCITONIN 1.1539568 1.1558753 1.183213 0.1440957

8 DDIMER 1.1438849 1.1462830 1.159712 0.1428999

9 TIBC 1.1103118 1.1294964 1.135731 0.1408072

10 FBS 1.1223022 1.1270983 1.164508 0.1405082

11 Muscle.Pain 1.0700240 1.0791367 1.085851 0.1345291

12 ESR 1.0724221 1.0791367 1.099760 0.1345291

13 NA. 1.0201439 1.0503597 1.061391 0.1309417

14 Taste.Smell 0.9942446 1.0000000 1.001918 0.1246637

15 CKD 0.9928058 0.9952038 1.000000 0.1240658

**S6 Table. Interaction Effects of Key Clinical Features with Age on Survival Outcomes**

.feature .class .interaction

<char> <fctr> <num>

1: Muscle.Pain:Age Alive 0.107484003

2: Muscle.Pain:Age Dead 0.107484003

3: Taste.Smell:Age Alive 0.048351279

4: Taste.Smell:Age Dead 0.048351279

5: CKD:Age Alive 0.004719824

6: CKD:Age Dead 0.004719824

7: O2sat.Ventilator:Age Alive 0.177557098

8: O2sat.Ventilator:Age Dead 0.177557098

9: NEUT:Age Alive 0.213951655

10: NEUT:Age Dead 0.213951655

11: NA.:Age Alive 0.100106824

12: NA.:Age Dead 0.100106824

13: P:Age Alive 0.220256603

14: P:Age Dead 0.220256603

15: FBS:Age Alive 0.116707323

16: FBS:Age Dead 0.116707323

17: ESR:Age Alive 0.110493978

18: ESR:Age Dead 0.110493978

19: LACTATE:Age Alive 0.138187846

20: LACTATE:Age Dead 0.138187846

21: PROCALCITONIN:Age Alive 0.121031985

22: PROCALCITONIN:Age Dead 0.121031985

23: Ferritin:Age Alive 0.200644927

24: Ferritin:Age Dead 0.200644927

25: TIBC:Age Alive 0.153809914

26: TIBC:Age Dead 0.153809914

27: DDIMER:Age Alive 0.132547047

28: DDIMER:Age Dead 0.132547047

.feature .class .interaction

**S7 Table. SHAP Values for The Selected Stacked Model**

feature class phi phi.var feature.value

1 Muscle.Pain Alive 0.00 0.00000000 Muscle.Pain=No

2 Taste.Smell Alive 0.00 0.00000000 Taste.Smell=No

3 CKD Alive 0.00 0.00000000 CKD=No

4 Age Alive 0.18 0.15061224 Age=-0.346153846153846

5 O2sat.Ventilator Alive 0.02 0.02000000 O2sat.Ventilator=0.166666666666667

6 NEUT Alive 0.08 0.07510204 NEUT=-0.967741935483871

7 NA. Alive 0.08 0.07510204 NA.=0.333333333333333

8 P Alive 0.04 0.03918367 P=-0.166666679912144

9 FBS Alive 0.00 0.00000000 FBS=0.574468085106383

10 ESR Alive 0.00 0.00000000 ESR=-0.333333333333333

11 LACTATE Alive -0.02 0.02000000 LACTATE=0.471042507437369

12 PROCALCITONIN Alive 0.02 0.02000000 PROCALCITONIN=0.249999986370889

13 Ferritin Alive 0.00 0.00000000 Ferritin=-0.224914434725919

14 TIBC Alive 0.04 0.08000000 TIBC=0.927007299270073

15 DDIMER Alive 0.00 0.00000000 DDIMER=0.669709543568465

16 Muscle.Pain Dead 0.00 0.00000000 Muscle.Pain=No

17 Taste.Smell Dead 0.00 0.00000000 Taste.Smell=No

18 CKD Dead 0.00 0.00000000 CKD=No

19 Age Dead -0.18 0.15061224 Age=-0.346153846153846

20 O2sat.Ventilator Dead -0.02 0.02000000 O2sat.Ventilator=0.166666666666667

21 NEUT Dead -0.08 0.07510204 NEUT=-0.967741935483871

22 NA. Dead -0.08 0.07510204 NA.=0.333333333333333

23 P Dead -0.04 0.03918367 P=-0.166666679912144

24 FBS Dead 0.00 0.00000000 FBS=0.574468085106383

25 ESR Dead 0.00 0.00000000 ESR=-0.333333333333333

26 LACTATE Dead 0.02 0.02000000 LACTATE=0.471042507437369

27 PROCALCITONIN Dead -0.02 0.02000000 PROCALCITONIN=0.249999986370889

28 Ferritin Dead 0.00 0.00000000 Ferritin=-0.224914434725919

29 TIBC Dead -0.04 0.08000000 TIBC=0.927007299270073

30 DDIMER Dead 0.00 0.00000000 DDIMER=0.669709543568465
