# Supplementary material for: Hybrid feature-selection and diversity-guided stacking framework for interpretable ensemble learning: Application to COVID-19 mortality prediction
Source: PLoS One. 2026 Apr 17;21(4):e0341198. doi: 10.1371/journal.pone.0341198 (PMC13089710; doi:10.1371/journal.pone.0341198)
Supplement: Attachment — Submitted filename: Response to Reviewer.docx [file pone.0341198.s003.docx]

**Response to Reviewers**

**Manuscript title:** *Improving COVID-19 Mortality Predictions: A Stacking Ensemble Approach with Diverse Classifiers*
**Journal:** PLOS ONE

Dear Dr. Huk,
We sincerely thank you and the reviewers for the careful evaluation of our manuscript and the constructive feedback. We have revised the paper accordingly, improving clarity, rigor, and presentation. We believe the manuscript is substantially improved as a result of these suggestions.

Below we provide a detailed, point-by-point response. Reviewer comments are shown in *italics*, followed by our responses. Changes are reflected in the **Revised Manuscript with Track Changes**.

**🔹 Editorial Requests**

1. *Language and presentation errors should be removed.*
   **Response:** We thank you for this note. The manuscript was carefully proofread for grammar, syntax, and clarity. Redundant definitions and awkward phrases were removed.
2. *Authors should reconsider if defining well known measures is needed (e.g. MCC, Cohen’s Kappa).*
   **Response:** We agree with this observation. Definitions of common evaluation metrics (MCC, Cohen’s Kappa, etc.) were removed, while retaining explanations only for less standard or newly applied measures.
3. *Balancing of usage of reference 1 and other references is highly advised.*
   **Response:** We acknowledge the over-reliance on a single reference in the original introduction. The literature review was expanded to include more diverse and recent works (2020–2025), reducing reliance on a single reference.
4. *It would be beneficial to add a flowchart that shows the stages of the proposed system and its operation.*
   **Response:** This is an excellent suggestion. A new flowchart (Figure 1) was added, summarizing the stages of data preprocessing, feature selection, model training, sub-model construction, and stacking.
5. *Limitations and biases of the study should be clearly discussed in a dedicated section.*
   **Response:** We agree that this is a critical point. A new “Limitations and Biases” section was added, addressing dataset representativeness, reliance on retrospective hospital data, possible measurement bias, and lack of external validation.
6. *Discussion of the generalizability of the model to non-Iranian population would increase the value of the study.*
   **Response:** A new paragraph was added (Discussion, section limitation and bias) explicitly addressing generalizability, noting cultural, healthcare system, and population differences, and suggesting the need for validation in international cohorts.
7. *Authors should not use unclear conditions in software/data availability statement (what is "reasonable" request?).*
   **Response:** The Data Availability statement was revised. We now specify that the dataset cannot be shared publicly due to patient confidentiality but can be accessed via a non-author institutional contact (Shahid Beheshti University Ethics Committee).
8. *Authors should consider that for larger samples also very tiny effects can be statistically significant. Effect size should be analysed (e.g., Wilcoxon R).*
   **Response:** Thank you for this important statistical point. Effect size analyses were added alongside statistical tests. We run Paired Wilcoxon with rstatix package and analyzed Effect size for paired test.
9. *Authors should consider applying post-hoc correction to p-values of many paired tests to control the family-wise error rate (FWER).*
   **Response:** We agree that controlling the family-wise error rate is crucial given the number of paired tests. we added these in methods.
10. *Data sharing statement must list a non-author institutional contact.*
    **Response:** Revised as requested; now lists the Ethics Committee office of Shahid Beheshti University of Medical Sciences with contact details.
11. *Your ethics statement should only appear in the Methods section.*
    **Response:** The ethics statement was removed from other sections and kept only in Method**s.**
12. *Supporting Information file [Data predictors.sav] not accessible.*
    **Response:** We apologize for the issue with the Supporting Information file. The file was removed.

**🔹 Reviewer #1**

We sincerely thank Reviewer #1 for their thorough and constructive feedback.

1. *Authors should clarify the novelty of the stacking approach compared to existing ensemble methods.*
   **Response:** Expanded in Introduction to emphasize novelty: integration of heterogeneous classifiers using multiple diversity measures, going beyond correlation-based stacking.
2. *Please justify the choice of 15 features selected via hybrid feature selection.*
   **Response:** We have clarified in the Methods section that the selection of 15 features was not arbitrary but was driven by both statistical and clinical considerations. Statistically, these features were identified through a hybrid feature selection pipeline integrating VIF, ANOVA, Sequential Backward Elimination, and Lasso regularization, which systematically removed redundant and low-contributing predictors. Clinically, the final 15 variables represent major physiological domains relevant to COVID-19 outcomes—such as inflammation (NEUT, ESR, Ferritin), hypoxia (O₂ saturation), metabolic disturbance (FBS, Lactate, Phosphorus), coagulation (D-dimer), and organ dysfunction (Procalcitonin, TIBC, CKD)—ensuring comprehensive coverage of mortality-related mechanisms while maintaining interpretability and avoiding overfitting. The resulting subset achieved a strong balance between model simplicity, predictive accuracy, and clinical relevance.
3. *Authors should explain why the Neural Network was chosen as the meta-learner over other options.*

**Response:** We have clarified that to integrate the predictions of sub-model sets, we applied a stacking ensemble framework using five meta-learners: Generalized Linear Model (GLM), Linear Discriminant Analysis (LDA), Random Forest (RF), Gradient Boosting Machine (GBM), and a Neural Network (NN). The choice of meta-learner was guided by both empirical testing and theoretical considerations of model diversity. Simpler linear meta-models, such as GLM or LDA, perform well when base classifiers are weakly correlated and exhibit near-additive relationships. However, when base learners capture heterogeneous and potentially non-linear patterns—as was the case in our ensemble of decision-tree, kernel-based, and regression models—more flexible meta-learners are required to effectively capture complex interactions among their predictions. Accordingly, the Neural Network meta-learner was selected as the optimal combiner due to its ability to model higher-order dependencies among base model outputs and its robustness in handling diverse feature distributions. Prior studies have shown that neural-network-based meta-learners can outperform linear or tree-based alternatives by capturing intricate non-linear relationships between model outputs, especially in biomedical and clinical prediction settings (An et al., 2020; Gupta et al., 2021; Kablan et al., 2023). We empirically verified this by cross-validation, where the NN meta-learner achieved superior discrimination and calibration compared to other stacking configurations.

4. *Provide more details on the handling of missing data beyond iterative imputation.*
   **Response:** We have expanded that the dataset used in this study had already undergone preprocessing using iterative multivariate imputation in Python’s Scikit-learn. This approach estimates missing values by sequentially modeling each variable as a function of the others, thus retaining complex inter-variable dependencies. It is widely recommended for clinical datasets where variables are correlated and missingness is assumed to be at random. Beyond imputation itself, the original data processing pipeline included the exclusion of categorical variables with missing values and continuous variables with more than 5% missingness, to minimize bias from excessive imputation. The imputed dataset was subsequently validated through distributional checks and correlation analyses to confirm plausibility and consistency. We have revised the Methods section (p. 16, lines 300) accordingly to clarify this process and to emphasize that the dataset used for modeling already incorporated robust and validated imputation procedures.
5. *Elaborate on the criteria for selecting the 16 base classifiers.*

**Response:** We appreciate the reviewer’s comment and have clarified the rationale for selecting our 16 base classifiers. The models were chosen based on three key criteria: (i) methodological diversity to ensure complementary error structures, (ii) prior evidence of strong performance in COVID-19 and biomedical prediction studies, and (iii) balance between interpretability and computational feasibility for clinical use.

This included linear models (e.g., GLM, Lasso, Ridge), probabilistic and instance-based methods (Naïve Bayes, KNN), and tree-based and ensemble learners (RF, XGBoost, GBM, AdaBoost). Such diversity enables the stacking framework to exploit heterogeneous strengths across model families. The revised Methods section now includes these selection criteria explicitly.

6. *The Literature Survey is weak. Authors should add the computational complexities and costs for all reviewed works. The authors should add the latest and most relevant works related to various types of cancers as [1] XAI-RACapsNet: Relevance aware capsule network-based breast cancer detection using mammography images via explainability O-net ROI segmentation; [2] dcnnbt: a novel deep convolution neural network-based brain tumor classification model; [3]brain tumor identification using data augmentation and transfer learning approach.; [4] u-net-based models towards optimal MR brain image segmentation.*
   **Response:** We have expanded the literature review to include recent studies addressing stacking ensemble models in cancer research and their computational implications. Specifically, we now discuss Mohammed et al. (2021), who demonstrated the computational feasibility and predictive superiority of a CNN-based stacking ensemble for multi-cancer classification; Wang et al. (2025), who applied a multimodal stacking framework for head-and-neck cancer prognosis achieving a C-index of 0.93; and Kwon et al. (2019), who systematically evaluated stacking configurations for breast cancer prediction, highlighting trade-offs between meta-learner choice and model complexity. These studies strengthen our justification for using stacking ensembles in clinical prediction tasks and align with our emphasis on balancing predictive power with computational efficiency. The corresponding paragraph has been added to the revised manuscript.
7. *Discuss the impact of class imbalance pre- and post-ROSE balancing.*
   **Response:** we added needed explanation.

*8. Authors should clarify how diversity metrics guided the selection of sub-model sets.
and 9. Justify the use of both pairwise and non-pairwise diversity measures.*

**Response:** We thank the reviewer for this insightful comment. We have clarified how diversity metrics were used to guide the construction of sub-model sets and justified the use of both pairwise and non-pairwise diversity measures. Specifically, pairwise measures were employed to identify and remove highly correlated or redundant classifiers, while non-pairwise metrics quantified the overall ensemble heterogeneity across multiple models. These metrics jointly ensured that retained sub-model sets maximized both predictive complementarity and diversity, improving the effectiveness of stacking.

*10. Explain why some stacking combinations did not improve accuracy.*

**Response:** We appreciate the reviewer’s insightful comment. Not all stacking combinations improved accuracy relative to their best-performing base classifiers. This is expected behavior and can arise for several reasons. First, when the base learners exhibit high prediction correlation or similar decision boundaries, the meta-learner receives redundant information and cannot extract additional signal. Second, if one base classifier (e.g., AdaBoost or RF) already dominates performance, its strong bias may overwhelm weaker learners, leading the stacked model to converge toward its predictions. Third, shallow or linear meta-learners (e.g., GLM, LDA) may be insufficient to capture the nonlinear interactions among base-model outputs. Conversely, deeper meta-learners (e.g., Neural Networks or GBM) improved accuracy in more heterogeneous sub-model sets (e.g., RF–XGB, NB–GBM), reflecting the importance of balancing diversity, complementarity, and meta-learner flexibility. *Provide confusion matrices.*

**Response:** Thank you for this suggestion. we revised table 6 and included *confusion matrices in it*.

11. *Discuss computational complexity and training time.*
    **Response:** We have added Training and Prediction Times for Single Models and Stacking Ensemble table (table 8) reporting approximate runtimes and scalability.
12. *Clarify SHAP computation for stacked model.*

**Response:** We have clarified in the Methods section that SHAP values were computed using a model-agnostic framework (iml package in R), applied directly to the final stacked ensemble (RF + XGB with NN meta-learner). In this setup, the entire stacked model was treated as a single predictive function, and SHAP decomposed the predicted probabilities into feature-level contributions of the original clinical variables. This approach ensures that the importance scores and interaction effects reflect how the ensemble as a whole integrates base-model predictions and clinical predictors, rather than attributing importance to individual base learners in isolation. We revised method and result part based on these comment.

13. *Explain moderate correlation between Ferritin, ESR, and TIBC clinically.*

**Response:** We expanded the explanation of the moderate correlations between Ferritin, ESR, and TIBC to clarify their clinical basis. These relationships reflect acute-phase inflammatory responses: Ferritin rises and TIBC falls during inflammation, while ESR increases due to elevated plasma fibrinogen and other acute-phase proteins. Thus, their moderate correlations capture biologically meaningful interactions consistent with systemic inflammation and disease severity.

1. *Discuss generalizability.*

**Response:** As noted in our response to the editor, we have expanded on this limitation in the Discussion section. We explicitly state that the model requires validation on external, diverse datasets before it can be considered for clinical use in other populations.

14. *Justify the absence of external validation despite a large dataset.*

**Response:** We acknowledge this as a primary limitation in our Discussion section. While a large independent internal test set was used, we did not have access to a suitable external dataset for validation at the time of the study. We have highlighted that external validation is a critical next step for future research.

15. *Explain the choice of robust scaling over other normalization methods.*

**Response:** We have clarified in the Methods section that Robust scaling handles skewed distributions and outliers better than alternatives.

16. *Discuss clinical practicality of predictors.*

**Response:** We have enhanced the Discussion to emphasize the clinical utility of the top predictors.

17. *Clarify hyper parameter tuning.*

**Response:** We have clarified in the Methods section that Model hyperparameters were optimized using grid search within the caret, guided by 10-fold cross-validation repeated 10 times to balance bias and variance. For algorithms where extensive tuning yielded negligible performance gains (e.g., GLM, LDA, CART, Naïve Bayes), default parameters were retained for computational efficiency. Parameter search ranges were defined based on prior literature and empirical recommendations for clinical prediction tasks. Final hyperparameters, shown in Table 1, represent the best-performing configurations in terms of cross-validated accuracy and AUC.

18. *Why traditional stacking underperformed.*

**Response:** We have addressed this in the Discussion that the traditional stacking approach underperformed relative to the diversity-guided ensemble primarily due to redundancy among correlated base learners. As shown by the high pairwise correlations, many models produced highly similar prediction patterns, offering limited incremental information to the meta-learner. This lack of diversity reduces the ensemble’s ability to generalize, as correlated errors can propagate through the stacking layer. In contrast, our diversity-based sub-model selection retained classifiers with complementary error profiles, enabling the meta-learner to better exploit non-overlapping decision boundaries. Consequently, the proposed approach achieved higher accuracy and stability by balancing predictive strength with heterogeneity among component models.

**🔹 Reviewer #2**

We thank Reviewer #2 for their detailed comments, which have helped us improve the manuscript significantly.

*1) The abstract lacks clarity. It should succinctly summarize the research objectives, methods, key findings, and implications in a structured manner.*

**Response:** We have restructured the abstract to follow a clear Objective, Methods, Results, and Conclusion format, improving its clarity and conciseness.

*2) The introduction lacks a comprehensive review of existing literature on COVID-19 mortality prediction. It should include a more thorough discussion of previous studies, highlighting gaps that the current research aims to fill.*

**Response:** The introduction review was expanded it now:

 **Reviews prior COVID-19 mortality studies** (traditional statistical and ML approaches, their limitations, imbalance, overfitting, bias, etc.).

 **Covers ensemble methods** (bagging, boosting, stacking) and clearly positions stacking as the next step.

 **Expands to computational cost and complexity** considerations — with multiple external domain references (oncology, radiomics, brain imaging).

 **Mentions methodological standards** (TRIPOD, calibration, effect-size reporting).

 **Clearly identifies gaps** (lack of diversity metrics, narrow base model sets, weak external validation, poor interpretability).

 **States the novelty and contribution** of your work (hybrid feature selection + diversity-guided stacking + interpretability).

*3) The manuscript should provide more details on the data collection process, including the reliability and validity of the data sources. Information on data preprocessing steps, such as handling missing values and outliers, is insufficient.*

**Response:** The “Data Sources and Ethical Considerations” and “Data Cleaning, Imputation and Data Normalization” stages were revised. They now:

 **Clearly described data collection** (source hospitals, timeframe, patient inclusion, ethics approval, RT-PCR confirmation, and clinical variables recorded).

 **Explained data reliability and validity** — by referencing Hatamabadi et al. (the epidemiological study describing the same cohort) and by mentioning ethical oversight.

 **Detailed preprocessing** — including exclusion criteria, missing value handling (thresholds, methods, rationale), and normalization.

 **Explained the rationale for iterative imputation** — describing why it’s preferable to mean or median imputation.

 **Explained the choice of robust scaling** — with reasoning tied to clinical data properties (non-normality, outliers).

*4)The rationale behind the chosen feature selection methods (VIF, ANOVA, SBE, Lasso) should be more explicitly stated. And 5) The manuscript should explain why these specific methods were selected over others and how they contribute to the study's objectives.*

**Response:** We appreciate the reviewer’s insightful comment. In the revised manuscript (Section 2.3 Feature Selection and Dimensionality Reduction), we have expanded the explanation to clarify the rationale for employing these four complementary feature selection methods. Specifically, we now emphasize that each technique was chosen to address distinct challenges inherent in high-dimensional clinical datasets—VIF to eliminate multicollinearity, ANOVA to identify discriminative variables, Sequential Backward Elimination (SBE) to iteratively refine feature subsets based on model performance, and Lasso regression to enforce sparsity and regularization. This hybrid approach ensures a balance between interpretability, computational efficiency, and predictive robustness.

*6) The selection of base models and meta-learners lacks justification. The manuscript should provide a clear rationale for choosing these particular algorithms, considering their strengths and limitations in the context of COVID-19 mortality prediction.*

**Response:** This has been clarified in the Methods that the combination of linear, tree-based, probabilistic, and neural methods was selected to reflect the heterogeneous nature of clinical COVID-19 data, which comprise continuous laboratory values, binary comorbidities, and ordinal symptom variables. Linear models (GLM, Lasso, Ridge) provided interpretability and baseline calibration; tree-based models (RF, XGBoost, GBM) captured non-linear feature interactions and threshold effects typical of clinical biomarkers; probabilistic models (Naïve Bayes, LDA) handled uncertainty in overlapping feature distributions; and the Neural Network component modeled complex, non-linear dependencies. For meta-learning, simpler models (GLM, LDA) were included for their transparency and stability, while ensemble-based (RF, GBM) and neural meta-learners were evaluated for their capacity to capture higher-order dependencies among diverse base model predictions. This design aligns with our study’s goal of achieving a clinically interpretable yet computationally efficient ensemble suited for mixed-type healthcare data.

*7) The diversity measures used to construct sub-model sets are not adequately explained. The manuscript should provide a detailed description of each measure (Disagreement, Yule's Q, Cohen's Kappa, etc. ) and its significance in enhancing model performance.*

**Response:** In the revised manuscript (Section 2.5 Constructing Sub-Model sets of Classifiers), we have expanded the explanation to:

**Diversity Measures**

To quantify heterogeneity among classifiers and guide sub-model selection, we employed both pairwise and non-pairwise diversity metrics, each capturing distinct aspects of predictive independence and complementarity.

**Pairwise metrics:**

– **Disagreement Measure:** quantifies the proportion of instances where two classifiers produce different predictions. Higher values indicate greater diversity and reduced redundancy.
– **Yule’s Q Statistic:** assesses the strength and direction of association between two classifiers’ predictions (range: –1 to +1). Lower absolute values denote weaker association and thus higher diversity.
– **Cohen’s Kappa:** evaluates inter-model agreement while adjusting for chance. Low or negative Kappa values suggest that classifiers make independent errors, which enhances ensemble robustness.
– **Double-Fault Measure:** measures the proportion of cases where both classifiers misclassify the same instance. Smaller values indicate complementary error patterns and reduced correlated failures.

**Non-pairwise metrics:**

– **Entropy Measure:** reflects the overall variability of predictions across all classifiers, ranging from 0 (perfect agreement, no diversity) to 1 (maximum diversity).
– **Kohavi–Wolpert Variance:** derived from error variance decomposition, it quantifies the dispersion of predictions across classifiers; higher values imply greater diversity and richer ensemble representation.

By combining pairwise and non-pairwise measures, we obtained a balanced evaluation of diversity—pairwise metrics were used to eliminate redundant learners, while non-pairwise metrics captured global variability across sub-model sets. This approach ensured complementary base models and improved generalization performance of the stacking ensemble.

*8) The choice of performance metrics (accuracy, sensitivity, specificity, etc.) should be justified. The manuscript should explain why these metrics are appropriate for evaluating the model's predictive capability in the context of COVID-19 mortality. And 9) The manuscript should explain why these tests are suitable for the current study design and data characteristics.*

**Response:** In the revised manuscript (Section 2. 7 Model performance), we have expanded the explanation to:

To compare the predictive performance of stacking approaches and sub-model sets, we evaluated models using standard **discrimination** and **calibration** metrics on an independent test dataset.

For discrimination, **accuracy, sensitivity, specificity, precision, F1-score, and Cohen’s Kappa** were computed. These metrics jointly assess overall correctness and class-specific performance—crucial in mortality prediction, where misclassifying high-risk patients (low sensitivity) may have serious clinical consequences, while excessive false alarms (low specificity) could strain resources. **Precision** and **F1-score** further balance false positives and negatives under class imbalance, and **Kappa** accounts for agreement beyond chance, providing a robust measure of classifier reliability.

The **area under the ROC curve (AUC)** quantified global discrimination across thresholds, whereas the **Matthews correlation coefficient (MCC)** offered a balanced assessment even with uneven class distributions—particularly relevant given lower mortality prevalence. **Calibration plots** were also used to assess the alignment between predicted probabilities and observed outcomes, reflecting the model’s reliability for clinical risk estimation. Statistical comparisons of model performance employed non-parametric tests. The **Wilcoxon signed-rank test** compared accuracy distributions across folds (with **Holm correction** for multiple comparisons) and reported **effect size (r = z/√N)** to indicate magnitude. **McNemar’s Test** examined classification differences between paired models, and **DeLong’s test** evaluated AUC differences under the null hypothesis of equal performance.

*10) The interpretation of results lacks depth. The manuscript should provide a more nuanced discussion of the findings, including potential limitations and biases in the data and models.*

**Response:** We have significantly expanded the Discussion section to provide a more nuanced interpretation of our findings, connecting them more directly to clinical practice and discussing potential data and model limitations in greater depth.

*11) The feature importance analysis should be more comprehensive. The manuscript should explore the interactions between features and their combined impact on mortality risk. And 12) The use of SHAP values for model interpretability is a strength, but the explanation of these values is insufficient. The manuscript should provide a clearer interpretation of SHAP values and their implications for clinical decision-making.*

**Response:** In the revised manuscript (Section 2. 7 Model performance and 3.6 7 Model Interpretation), we have revised the explanation of the SHAP analysis to make it more accessible to a clinical audience. We now provide clearer interpretations of what the SHAP values signify for an individual patient's predicted risk, thereby enhancing the model's transparency.

*13) The discussion section should be more comprehensive, addressing the study's implications for clinical practice, future research directions, and potential societal impact. The conclusion should be more robust, summarizing the key findings, limitations, and future research needs.*

**Response:** Both the Discussion and Conclusion sections have been thoroughly revised to better synthesize our findings, address the study’s limitations, and outline clear directions for future research, including the critical need for external validation and prospective studies.

*14) The manuscript contains numerous obvious formatting and grammatical issues. The authors should carefully proofread their submission to improve its writing quality. For example, there are many evident errors in the references list.*

**Response:** We sincerely apologize for these errors. The entire manuscript has undergone a thorough professional copyediting process to correct all formatting, spelling, and grammatical mistakes, including a careful review of the reference list.

**🔹 Reviewer #3**

We thank Reviewer #3 for their helpful suggestions for improving the manuscript's structure and clarity.

*1- The abstract needs a brief introduction to the research gap before proceeding to state what has been proposed.*

**Response:** We agree. The abstract has been revised to begin with a concise introductory sentence that establishes the research gap before detailing our proposed method and findings.

*2- The percentage of using reference 1 in the introduction is very high, so it is preferable to add other references and diversify the paragraphs by diversifying their references. And 3- The related works listed do not cover a large period of time, such as between 2020-2025.*

**Response:** We acknowledge this issue. The introduction has been revised it now:

 **Reviews prior COVID-19 mortality studies** (traditional statistical and ML approaches, their limitations, imbalance, overfitting, bias, etc.).

 **Covers ensemble methods** (bagging, boosting, stacking) and clearly positions stacking as the next step.

 **Expands to computational cost and complexity** considerations — with multiple external domain references (oncology, radiomics, brain imaging).

 **Mentions methodological standards** (TRIPOD, calibration, effect-size reporting).

 **Clearly identifies gaps** (lack of diversity metrics, narrow base model sets, weak external validation, poor interpretability).

 **States the novelty and contribution** of your work (hybrid feature selection + diversity-guided stacking + interpretability).

*4- The technical basics used are not separated from the stages of work of the proposed algorithm.*

**Response:** This is an excellent structural suggestion. A new flowchart **(Fig. 1)** was added. As illustrated in **Fig. 1**, this study employed a multi-stage framework for mortality risk prediction, consisting of both technical foundations and novel algorithmic contributions. The overall workflow can be divided into two layers:

1. **Foundational (Technical) Stages** – encompassing data preprocessing, feature normalization, and base model training using established machine learning procedures.
2. **Proposed Algorithmic Stages** – introducing a novel diversity-guided stacking ensemble framework that systematically integrates hybrid feature selection, explicit diversity quantification, and multi-meta-learner evaluation to optimize predictive performance, interpretability, and computational efficiency.

*5- Add a flowchart that shows the stages of the proposed system's operation.*

**Response:** We agree that a flowchart would be highly beneficial. A new flowchart (Figure 1) was added, summarizing the stages of data preprocessing, feature selection, model training, sub-model construction, and stacking.

We believe these revisions have significantly strengthened our manuscript. We are grateful for the reviewers’ and editor’s valuable feedback and hope that the revised version meets the journal’s standards.

Sincerely,
Farideh Mohtasham
On behalf of all co-authors
