# Supplementary material for: Hybrid feature-selection and diversity-guided stacking framework for interpretable ensemble learning: Application to COVID-19 mortality prediction
Source: PLoS One. 2026 Apr 17;21(4):e0341198. doi: 10.1371/journal.pone.0341198 (PMC13089710; doi:10.1371/journal.pone.0341198)
Supplement: Attachment — Submitted filename: Response_to_Reviewer_auresp_2.docx [file pone.0341198.s004.docx]

**Response to Reviewers**

**Manuscript title:**

*A Hybrid Feature-Selection and Diversity-Guided Stacking Framework for Interpretable Ensemble Learning: Application to COVID-19 Mortality Prediction (PONE-D-25-18852R1)*

Dear Editor and Reviewers,

We thank the Academic Editor and the reviewers for their constructive and detailed feedback. We have revised the manuscript thoroughly based on the comments. All revisions have been incorporated in both the “Revised Manuscript with Track Changes” and the clean version.

Below we provide a point-by-point response.

**GENERAL COMMENTS**

• All reviewers’ and editor’s concerns have been fully addressed.
• The entire manuscript has undergone comprehensive English language editing to remove grammatical, stylistic, and formatting issues.
• Presentation issues in figures, tables, and references have been corrected.
• The data standardization section has been rewritten for clarity, explicitly explaining the use of Robust Scaler and distinguishing standardization from normalization.

**RESPONSE TO ACADEMIC EDITOR**

**1. “Multiple language problems should be removed.”**

✓ The manuscript has been fully reviewed and professionally edited.
✓ Ambiguous, grammatically incorrect, or unclear sentences have been rewritten throughout the text.
✓ Reference formatting has been standardized.

**2. “Multiple presentation problems need to be corrected.”**

All the issues noted by Reviewer #4 have been addressed individually (details below).

**3. “Data standardisation process should be made clear.”**

- Explained the use of **Robust Scaler** as a *standardization* method
- The confusing term “robust_scalar” was corrected to “Robust Scaler.”

**RESPONSE TO REVIEWER #4**

**1. Language Problems**

**1.1–1.5 Incorrect capitalization in references**
✓ Corrected to:

- European Journal of Haematology
- BMJ
- IEEE Access
- Information Fusion
- CRC Press

**2. Presentation Problems**

**2.1 Invalid citation [90,92]**
✓ Fixed by correcting the missing or mismatched references.

**2.2 Incomplete reference [67]**
✓ Updated with full bibliographic information.

**2.3 Invalid URL in reference [58]**
✓ Corrected or replaced with valid official reference source.

**2.4 Missing reference details [59]**
✓ Completed with full citation.

**2.5 Old reference with capitalization issues**
✓ Citation corrected to sentence case.

**2.6 Reference [46] incomplete**
✓ Updated with complete information.

**2.7 Reference formatting inconsistent**
✓ Entire reference list reformatted.

**2.8 Figure 1: Inaccurate connector location**
✓ We clarified the figure by ensuring that the connection arrows to the Pairwise measures (Disagreement, Yule’s Q, Cohen’s Kappa, Double-Fault) and non-pairwise metrics (Entropy, Kohavi–Wolpert) are precisely aligned and visually consistent.

**2.9 Figure 7 missing whiskers**
✓ As requested, we regenerated the performance figure using per-resample performance metrics from repeated 10-fold cross-validation on train data. We computed Accuracy, Sensitivity, Specificity, Precision, F1, and MCC for every resample and plotted mean ± SD (whiskers) for each stacking model. This revised figure is now included in the manuscript.

**2.10 Inconsistent capitalization of abbreviations (RF, NB, etc.)**
✓ Standardized throughout text, tables, and figures.

**2.11 Figure titles unclear regarding dataset**
✓ All figure captions now specify whether results are from training, cross-validation, or testing datasets.

**2.12 Table 6 too wide**
✓ Reformatted to reduce empty space.

**2.13 Table 5 missing header label**
✓ Header updated.

**2.14 Table 3 font size inconsistent**
✓ Font standardized.

**3. Other Problems**

**3.1 Incorrect function name “robust_scalar”**
✓ Corrected to “Robust Scaler”

**3.2 Clarification: standardization vs normalization**
✓ A rewritten paragraph now states that Robust Scaler performs **standardization**, not normalization.

We sincerely thank the reviewers and editor for their constructive feedback, which has strengthened the clarity, correctness, and overall quality of the manuscript.

We hope the revisions meet the publication criteria, and we respectfully request reconsideration for acceptance.

Sincerely,
Farideh Mohtasham
(on behalf of all authors)
